# Spatial associations of Hansen’s disease and schistosomiasis in endemic regions of Minas Gerais, Brazil

**DOI:** 10.1371/journal.pntd.0012682

**Published:** 2024-12-26

**Authors:** Jessica L. Stephens, Lucia A. O. Fraga, José A. Ferreira, Laura De Mondesert, Uriel Kitron, Julie A. Clennon, Jessica K. Fairley

**Affiliations:** 1 Department of Epidemiology, Rollins School of Public Health, Emory University, Atlanta, Georgia, United States of America; 2 Universidade Federal Juiz de Fora–Campus Governador Valadares, Governador Valadares, Minas Gerais, Brazil; 3 Universidade Vale do Rio Doce, Governador Valadares, Minas Gerais, Brazil; 4 Faculdade da Saúde e Ecologia Humana (FASEH), Vespasiano, Minas Gerais, Brazil; 5 Hubert Department of Global Health, Rollins School of Public Health, Emory University, Atlanta, Georgia, United States of America; 6 Department of Environmental Sciences, Emory University, Atlanta, Georgia, United States of America; 7 Division of Infectious Diseases, Department of Medicine, Emory University School of Medicine, Atlanta, Georgia, United States of America; Institute of Continuing Medical Education of Ioannina, GREECE

## Abstract

**Background:**

Brazil has the second highest case count of Hansen’s disease (leprosy, HD), but factors contributing to transmission in highly endemic areas of the country remain unclear. Recent studies have shown associations of helminth infection and leprosy, supporting a biological plausibility for increased leprosy transmission in areas with helminths. However, spatial analyses of the overlap of these infections are limited. Therefore, we aimed to spatially analyze these two diseases in a co-endemic area of Minas Gerais, Brazil, in order to identify potential epidemiologic associations.

**Methods:**

An ecological study using public health surveillance records and census data was conducted to investigate whether the occurrence of HD -and specifically multibacillary (MB) disease- was associated with the presence of schistosomiasis in a community of 41 municipalities in eastern Minas Gerais, Brazil from 2011 to 2015. Multivariate logistic regression and spatial cluster analyses using geographic information systems (GIS) were performed.

**Results:**

The average annual incidence of HD in the study area was 35.3 per 100,000 while *Schistosoma mansoni* average annual incidence was 26 per 100,000. Both HD and schistosomiasis were spatially distributed showing significant clustering across the study area. Schistosomiasis was present in 10.4% of the tracts with HD and thirteen high-high clusters of local bivariate autocorrelation for HD and schistosomiasis cases were identified. A multivariate non-spatial analysis found that census tracts with MB disease were more likely to have schistosomiasis when adjusted for population density, household density, and household income (aOR = 1.7, 95% CI 1.0, 2.7). This remained significant when accounting for spatial correlation (aOR = 1.1, 95% CI (1.0, 1.2)).

**Conclusion:**

We found clustering of both HD and schistosomiasis in this area with some statistically significant overlap of multibacillary HD with *S*. *mansoni* infection. Not only did we provide an effective approach to study the epidemiology of two endemic neglected tropical diseases with geographic spatial analyses, we highlight the need for further clinical and translational studies to study the potential epidemiologic associations uncovered.

## Author summary

As cases of Hansen’s disease decrease globally, areas with high endemicity still struggle to control infections. In Brazil, which has the second highest burden of disease globally, new measures are necessary to reach elimination. Not only are geographic information systems (GIS) important tools to describe the heterogeneity of infectious diseases on a finer scale, it can also be used to study the overlap of two different endemic infections. Due to several studies showing potential associations between helminth infection and Hansen’s disease, this study explores a co-endemic helminth infection *(Schistosoma mansoni)* as a potential factor in the continued transmission of leprosy. Our objective was to spatially analyze these two diseases and identify potential epidemiologic associations in 41 municipalities in the state of Minas Gerais, Brazil. Our study found the risk of MB disease was over 1.5 time greater in census tracts with reported schistosomiasis than in tracts without, adjusted for socioeconomic and environmental factors. We also identified several clusters of schistosomiasis and MB disease. While these findings can provide immediate data for control programs to utilize, it also calls for further studies on the interplay of co-infections in the manifestations of HD that may shed light on ongoing transmission in highly endemic areas.

## Introduction

Brazil has the second highest burden of Hansen’s disease, (leprosy) in the world, after India. While cases of Hansen’s disease (HD) have decreased over the last 20 years, obstacles have limited progress in interrupting transmission of *Mycobacterium leprae*, including a long incubation period, stigma, and suboptimal public health measures [1]. Since differences in *M*. *leprae* virulence are limited, host factors are thought to explain the diverse clinical forms of Hansen’s disease (HD) [2,3]. Due to overlapping factors related to poverty, helminth infections are commonly endemic in areas where HD is prevalent [4–6]. These diseases share many of the same geographic areas including in endemic areas of Brazil [5]. Growing, though still limited, evidence suggest that the immune derangements of helminth infections can reduce the cell-mediated immune control of mycobacterial infections like tuberculosis and leprosy [7–10].

Helminth infections have been associated with both total leprosy cases as well as the more transmissible, lepromatous, end of the HD disease spectrum [7,10,11]. In Diniz et al, Th2 cytokine levels were more common in those with HD- soil-transmitted helminth co-infection, especially those at the lepromatous end of the spectrum [7]. Multibacillary leprosy, especially lepromatous disease, is more commonly associated with a Th2 immune response, during which the innate Th1 response is downregulated [1,12]. Helminth infections are also characterized by a shift to the Th2 immune response, possibly providing a mechanistic explanation for immune deregulation that can make patients more susceptible to the more infectious form of HD, clinically characterized as multibacillary disease, MB, and thus sustain a continuous source of transmissible *M*. *leprae* infection in the community [13,14]. A pilot study in Vespasiano, Minas Gerais, Brazil suggested a potential spatial association between HD and schistosomiasis, an important helminth infection. This study found a relative risk of 6.8 (CI = 1.5, 31.6) of HD in neighborhoods with reported schistosomiasis, independent of population density and purchasing power per capita [15]. In addition, we also found that individuals with HD were more likely to be infected with *S*. *mansoni* then household contacts in a case-control study from the same region [16].

Understanding the epidemiology of HD and schistosomiasis in an area of high HD incidence may shed light on potential drivers in the ongoing transmission. Geographic Information Systems (GIS), therefore, can be used to investigate how these two infections overlap in the same geographic area. In fact, GIS have been used to study both HD and schistosomiasis in Brazil and in other endemic areas [4,17,18], but studies on their co-occurrence are limited outside of the small study from our group [15]. Spatial and temporal studies of HD in endemic areas in Brazil using surveillance data collected by the Information System for Notifiable Diseases [Sistema Nacional de Agravos de Notificação] (SINAN) have applied spatio-temporal cluster analysis and spatial regression [18–23]. Our objective, therefore, was to spatially analyze these two diseases in a co-endemic area of Minas Gerais, Brazil in order to build on our findings from our pilot study in Vespasiano and better describe spatial associations of the two infections [15]. Findings can then be used to investigate public health interventions and further mechanistic studies on the impact of co-infections on disease transmission.

## Methods

### Ethics statement

This study was approved by the Institute of Health Sciences Research Ethics Committee from the Universidade Vale do Rio Doce (CAAE: 56700816.3.0000.5157 CEP) and the Emory University Institutional Review Board (IRB: IRB00087575).

### Study Area

**Fig 1:**
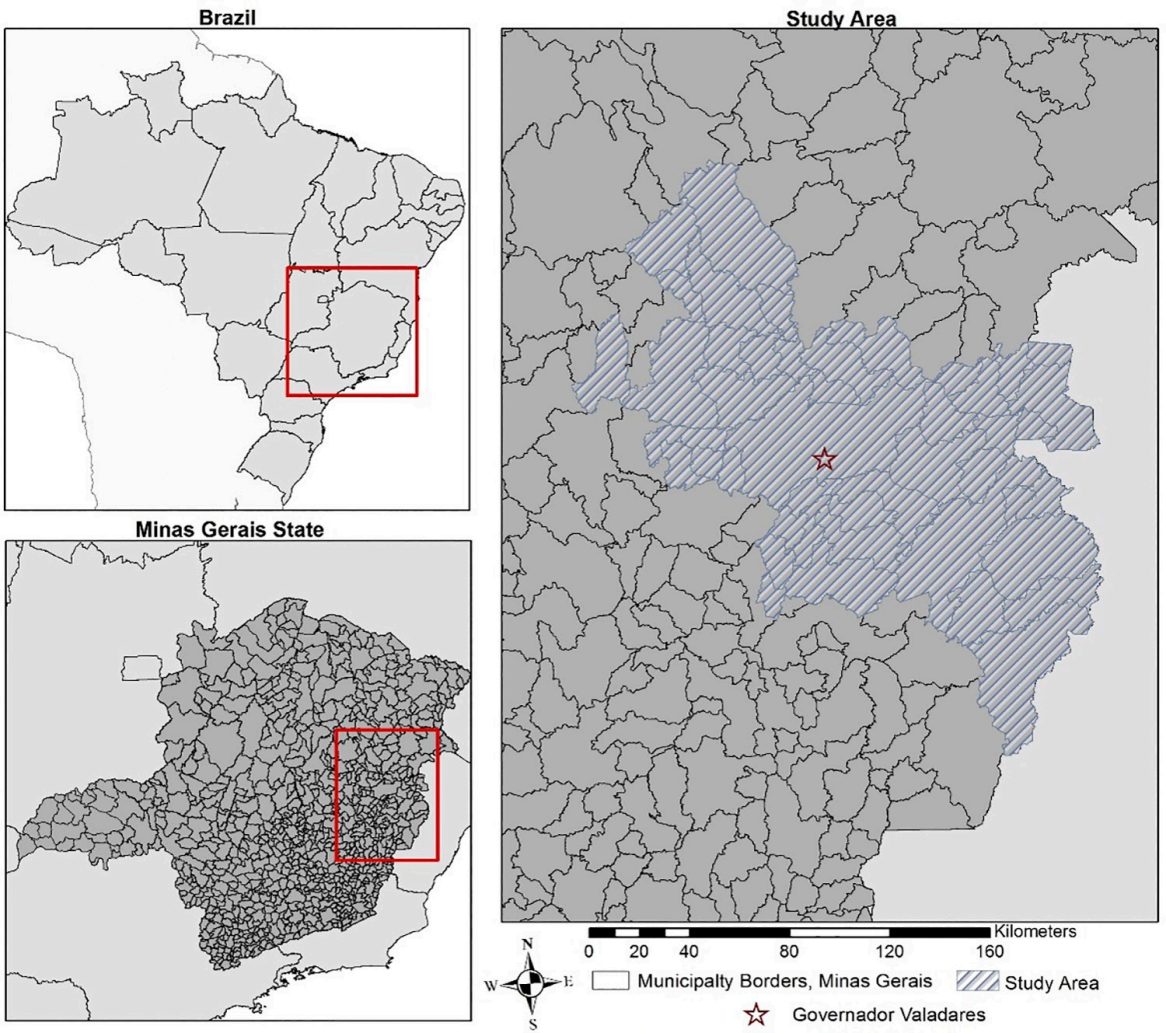
Study Area: 41 Municipalities in Minas Gerais, Brazil. Map produced in ArcGIS 10.4 (ESRI, Redlands, CA, USA) using the spatial reference SIRGAS 2000 UTM Zone 24S30. (https://spatialreference.org/ref/epsg/31984/).

This study was conducted in the Brazilian state of Minas Gerais, Brazil’s second most populous state, fourth largest by area and with the third highest gross domestic product. The area is endemic for both HD and *Schistosoma mansoni* infection. The specific region is displayed in Fig 1 and surrounding Governador Valadares (21,100 km^2^). This region reports data to Governador Valadares, and comprises 41 municipalities and 1,272 census tracts (Fig 1). The population of the study area in 2010 was 610,618 people [24]. In the study area, the average annual incidence of Hansen’s disease was 35.3 per 100,000 (7.3% of cases were found in children 15 years old and under, 55.3% were classified as MB disease). Schistosomiasis annual incidence was 2.6 per 10,000 (26 per 100,000).

### Data sources and methods

In Brazil, HD and schistosomiasis (*Schistosoma mansoni* infection) are included in the Information System for Notifiable Diseases (Sistema Nacional de Agravos de Notificação, SINAN) as reportable diseases. HD is a compulsory notifiable disease; thus, all patients detected through surveillance are registered with clinical, demographic, and address data. Notification of schistosomiasis is recommended by the Schistosomiasis Control Program [Programa de Controle da Esquistossomose] and SINAN reporting has been mandatory since 1998 [25]. The Regional Health Managementprovided detailed data from SINAN including residential addresses, demographic and epidemiological variables (age, gender and operational classification) for the years 2011–2015 to do the analyses while names were not provided to maintain confidentiality. Patient recorded addresses were geocoded using Google Earth Pro version 7.1.7 using two algorithms, however, no maps were made using address or point data to preserve confidentiality.

To control for other potential sociodemographic factors that have been associated with HD transmission in prior studies [26,27], other census level demographic variables were obtained from the 2010 census, available through the Brazilian Institute of Geography and Statistics [Instituto Brasileiro de Geografia e Estatıstica 2011] [24] (IBGE). These variables included population per census tract (residents in permanent private households), household density (average number of residents in private households) and income (nominal average monthly income of persons responsible for permanent private households). Household density was categorized as high (above 3) or low (3 or less) split. Income and population density were ranked by tertiles.

### Spatial data analysis

Spatial analysis was conducted using individual georeferenced patient location and cases aggregated to the census tract. At the georeferenced point level, we described individual cases and clusters of HD, MB disease (as a marker of the most infectious form of Hansen’s disease) and schistosomiasis. At the aggregated level, we compared average annual incidence of HD cases per 100,000, the average annual incidence of multibacillary HD cases per 100,000, and the average annual incidence of schistosomiasis cases per 10,000. These measures (per 100,000 vs per 10,000) were chosen due to accepted convention in the literature for these infections. All maps were produced in ArcGIS 10.4 (ESRI, Redlands, CA, USA) using the spatial reference SIRGAS 2000 UTM Zone 24S [28] (https://spatialreference.org/ref/epsg/31984/).

For georeferenced point level data, methods to address spatial randomness of each infection respectively, as well as compare multibacillary (MB) to paucibacillary (PB) disease distribution were employed. Ripley’s K-function was used to assess global spatial clustering considering a range of distance 50 m to 5,000 m or 17,500 m (univariate or bivariate, respectively) with distance lags of 500 m (with edge correction) [29]. This range was chosen to include a fine scale due to increased risk of infections for household contacts and neighbors of HD patients, with the risk inversely decreasing with increasing distances [30,31]. ArcGIS was used to calculate Ripley’s K-function for individual outcomes (HD, MB disease and schistosomiasis, respectively) and Point Analysis, Spatial Statistics and Geographic Exegesis (PASSaGE v2) was used to assess dichotomous data using bivariate K-function (MB disease vs PB disease) [32].

Kulldorff’s spatial scan statistics were also applied to detect the most likely high-risk clusters of individual cases considering a distribution of controls (Bernoulli model). For the pure spatial analysis, this statistical technique uses a flexible elliptical geographic scanning window to included different sets of neighbors, ranging from the minimum distance between points to half the width of the study area. This method was used to test the null hypothesis of constant risk between points [33]. StatScan version 9.4.4 (Harvard Medical School, Boston, USA) was used to perform Kulldorff’s spatial scan statistics.

Data were then aggregated to census tract to compare spatial patterns between infections. To minimize effect of small numbers on statistical instability, we performed spatial empiric Bayesian smoothing to estimate smoothed incidence between contiguous areas (using a queen spatial weigh matrix [34]. Anselin’s local indicator of spatial association (LISA) was applied to characterize areas with statistically significant (p<0.05) spatial autocorrelations for univariate (all HD incidence, MB incidence alone, schistosomiasis incidence) and bivariate associations (all HD incidence with schistosomiasis, MB incidence alone with schistosomiasis) [35]. Finally, Kulldorff’s spatial scan statistics were again applied to detect the most likely high-risk clusters of cases per census tract considering the rest of the population as controls to identify adjacent census tracts least consistent with the hypothesis of constant risk. The following software were used for these spatial analyses: GeoDa 1.6 to calculate spatial weight matrix, spatially empirical Bayes incidence per census tract and LISA; and SatScan to perform Kulldorff’s spatial scan statistics [35,36].

### Logistic regression data analysis

Logistic regression was conducted to examine the association of census tract incidence of schistosomiasis with incidence of total HD and then MB alone controlling for average household density, population density and average household income using SAS 9.4 (Cary, NC). Models were created at the aggregated level for both HD and MB (presence/absence) as the outcome, respectively. Census tract level variables obtained from the 2010 census (average household density, population density and average household income as defined above) were assigned to individual cases based on census tract of residence. Models were assessed for collinearity and effect modification. Final logistic regression models were then assessed for global spatial autocorrelation using RStudio (Version 3.3.1). Both a spatial lag and spatial error model was applied to models found to have significant spatial autocorrelations using a Moran’s I test. If both alternative models improved the original model the final spatially adjusted model was chosen by comparing performance parameters R-squared, Log likelihood, AIC (Akaike Info Criterion).

## Results

### Study population

The data included reported cases of HD (n = 1,078) and schistosomiasis (n = 783) from January 1, 2011 to December 23, 2015. In the study area, the average annual incidence of Hansen’s disease was 35.3 per 100,000 (7.3% of cases were found in children 15 years old and under, 55.3% were classified as MB disease). Schistosomiasis annual incidence was 2.6 per 10,000 (26 per 100,000). There were 755 cases of HD (83.0% of cases with street addresses) and 313 cases of schistosomiasis (95.7% of cases with street addresses) that had sufficient data to be mapped through geocoding. Most cases not geocoded were due to missing street addresses (58.2% of the schistosomiasis cases and 16.7% of the HD cases). A flowsheet of inclusion can be found in the supplemental materials **(S1 Fig).** Georeferenced data were compared to the original dataset and there was no significant difference between descriptive and clinical statistics for the two, and the percent representation of cases by municipality was similar between the datasets (**S2 and S3 Figs**).

Demographic and clinical variables of the HD cases are outlined in **Table 1**. Of the total georeferenced HD cases, 388 (51.4%) were MB and 387 were male (51.3%); 65 (8.6%) were pediatric, (**Table 1**). The mean age was 47.1 years (SD 19.6) Seventy four (9.8%) of geocoded individuals with HD lived in census tracts with reported schistosomiasis. Based on statistics abstracted from census records for use in the model, in tracts with HD, the average monthly income was $974.8 Brazilian Reais, average household density was 3.2 people per household, and average population density was 5,492 people per square kilometer.

**Table 1:** Descriptive Characteristics of Incident Cases of Hansen’s Disease in Minas Gerais, 2011–2015 (n = 755).

Variable	Mean (SD) or N (%)
**Hansen’s Disease Operational Classification**	
Multibacillary	388 (51.4)
Paucibacillary	367 (48.6)
**Residence in Census Tracts with reported Schistosomiasis**	74 (9.8)
**Sex**	
Male	387 (51.3)
Female	368 (48.7)
**Age (continuous)**	47.1 (19.6)
**Age (by 15 year category)**	
0–15	65 (8.6)
16–30	89 (11.8)
31–45	117 (23.4)
46–60	218 (28.9)
61–75	155 (20.5)
76–93	51 (6.5)

### Individual level spatial analysis

Looking at Hansen’s disease spatially, Ripley’s k-function detected significant *global* clustering of individual cases for both MB and PB Hansen’s disease diseases, starting from 500 meters to the maximum distance tested (**S4 Fig**). However, when looking for *locally* defined clustering, Kulldorff’s spatial scan analysis did not detect any local clustering for all HD cases, MB disease, or schistosomiasis.

### Census tract description

**Fig 2:**
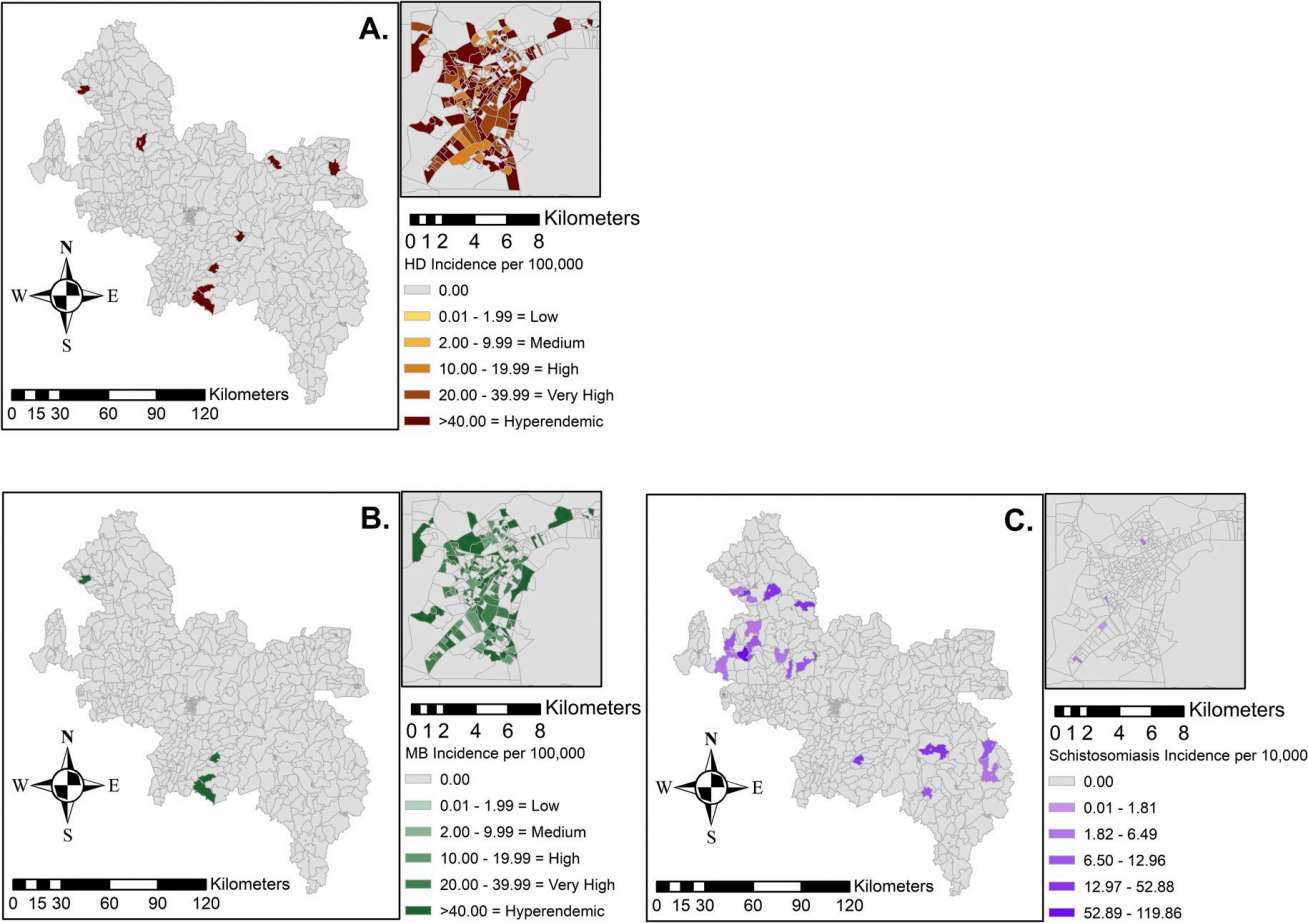
Univariate Spatial Analysis: Raw Annual Incidence of Aggregated census tract level spatial analysis for all HD cases, MB disease and Schistosomiasis, respectively (A, B, C) with Governador Valdares inset. Maps were produced in ArcGIS 10.4 (ESRI, Redlands, CA, USA) using the spatial reference SIRGAS 2000 UTM Zone 24S30 (https://spatialreference.org/ref/epsg/31984/).

**Fig 3:**
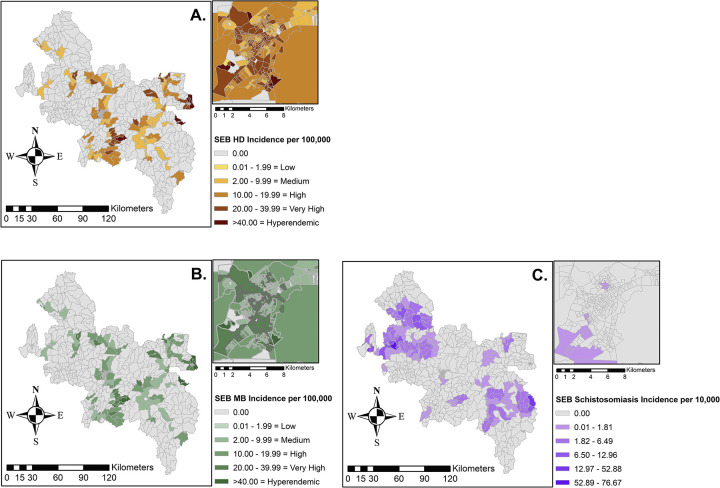
Univariate Spatial Analysis: Spatially Empirical Bayesian approach to smoothing of aggregated census tract level spatial analysis for all HD cases, MB disease and Schistosomiasis, respectively (A, B, C) with Governador Valadares Inset. Maps were produced in ArcGIS 10.4 (ESRI, Redlands, CA, USA) using the spatial reference SIRGAS 2000 UTM Zone 24S30 (https://spatialreference.org/ref/epsg/31984/).

In the raw data, HD was reported in 355 / 1,272 tracts (27.9%), MB diseases were present in 247 tracts (19.4%), and schistosomiasis was present in 107 census tracts (8.4%) (**Fig 2A–2C).** Hyperendemic HD (defined as >40 annual cases / 100,000) existed in 210 tracts. Taking into account their closest neighbor with local Bayesian analysis, HD was extrapolated to 723 tract and MB disease to 633 tracts **(Fig 3).** As with the raw data, 210 tracts were hyperendemic for HD in the SEB analysis; however, the maximum average annual incidence shrunk to 202.9 cases per 100,000 people from 500 cases per 100,000 in the raw data (**Fig 2A–2C**).

### Census tract spatial analysis

**Fig 4:**
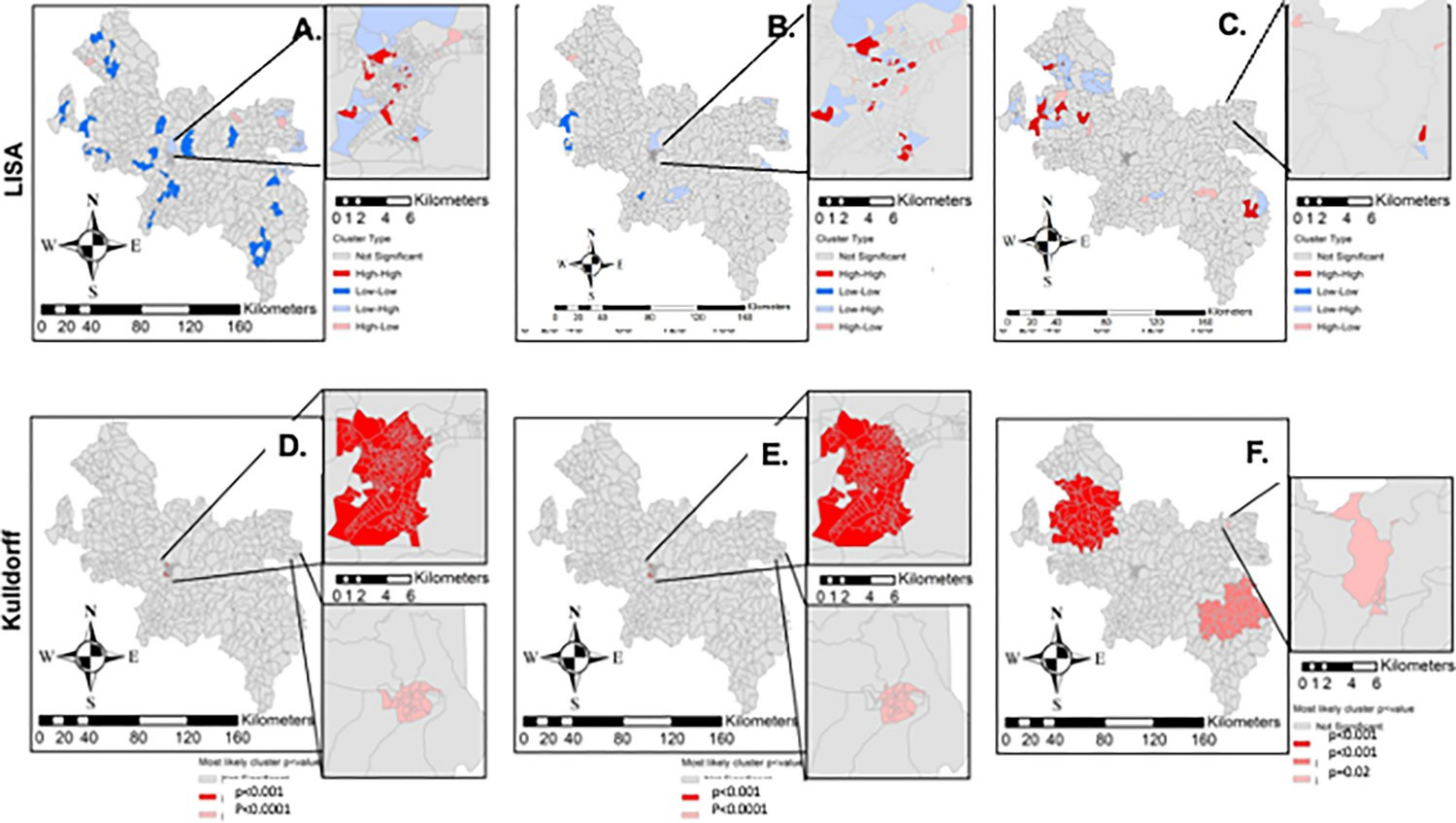
Univariate Spatial Analysis: Local indicator of Spatial Autocorrelation (LISA), and Kulldorff’s Spatial Scan Statistics most likely clusters of aggregated census tract level spatial analysis for all HD cases, MB disease and schistosomiasis, respectively (A, B, C) with either Governador Valadares urban area Insets or other Cluster Insets for better visualization. Maps were produced in ArcGIS 10.4 (ESRI, Redlands, CA, USA) using the spatial reference SIRGAS 2000 UTM Zone 24S30 (https://spatialreference.org/ref/epsg/31984/).

LISA detected significant associations (p<0.05) between census tracts with high incidence rates to their neighbors (high-high). Visualized in **Fig 4,** 50, 37, and 43, LISA clusters were identified for all HD cases, MB cases, and schistosomiasis, respectively (**Table 2**). Kulldorff’s spatial scan statistics also indicated the most likely clusters of HD, MB, and schistosomiasis (**Fig 4** and **Table 3**). Some similarities in LISA and Kulldorff’s clustering were identified for HD and MB disease, as expected. Tight clusters were identified for HD, MB, and schistosomiasis.

**Table 2:** Spatial autocorrelation of HD, MB disease and schistosomiasis separately (univariate), and then compared to each other as indicated (bivariate).

LISA Clusters	High Clusters	Low Clusters
**Univariate**		
Total HD Cases	50	27
MB HD	37	3
Schistosomiasis	43	4
**Bivariate**		
HD/Schistosomiasis	13	3
MB/Schistosomiasis	8	3

**Table 3:** Kulldorff’s spatial scan of most likely clusters of total HD, MB disease And schistosomiasis.

Cluster Number	Type of Infection (s)	Number of Census Tracts	Observed Cases	Expected Cases	Relative risk	P-value
1	Hansen’s Disease (MB/PB)	289	189	81.3	3.8	<0.001
2	Hansen’s Disease (MB/PB)	28	24	7.9	3.2	<0.001
1	MB Disease	265	130	52.0	4.2	<0.001
2	MB Disease	25	18	4.9	3.9	<0.001
1	Schistosomiasis	108	1449	9.2	9.0	<0.001
2	Schistosomiasis	82	25	7.0	4.4	<0.001
3	Schistosomiasis	9	7	0.8	9.7	0.017

**Fig 5:**
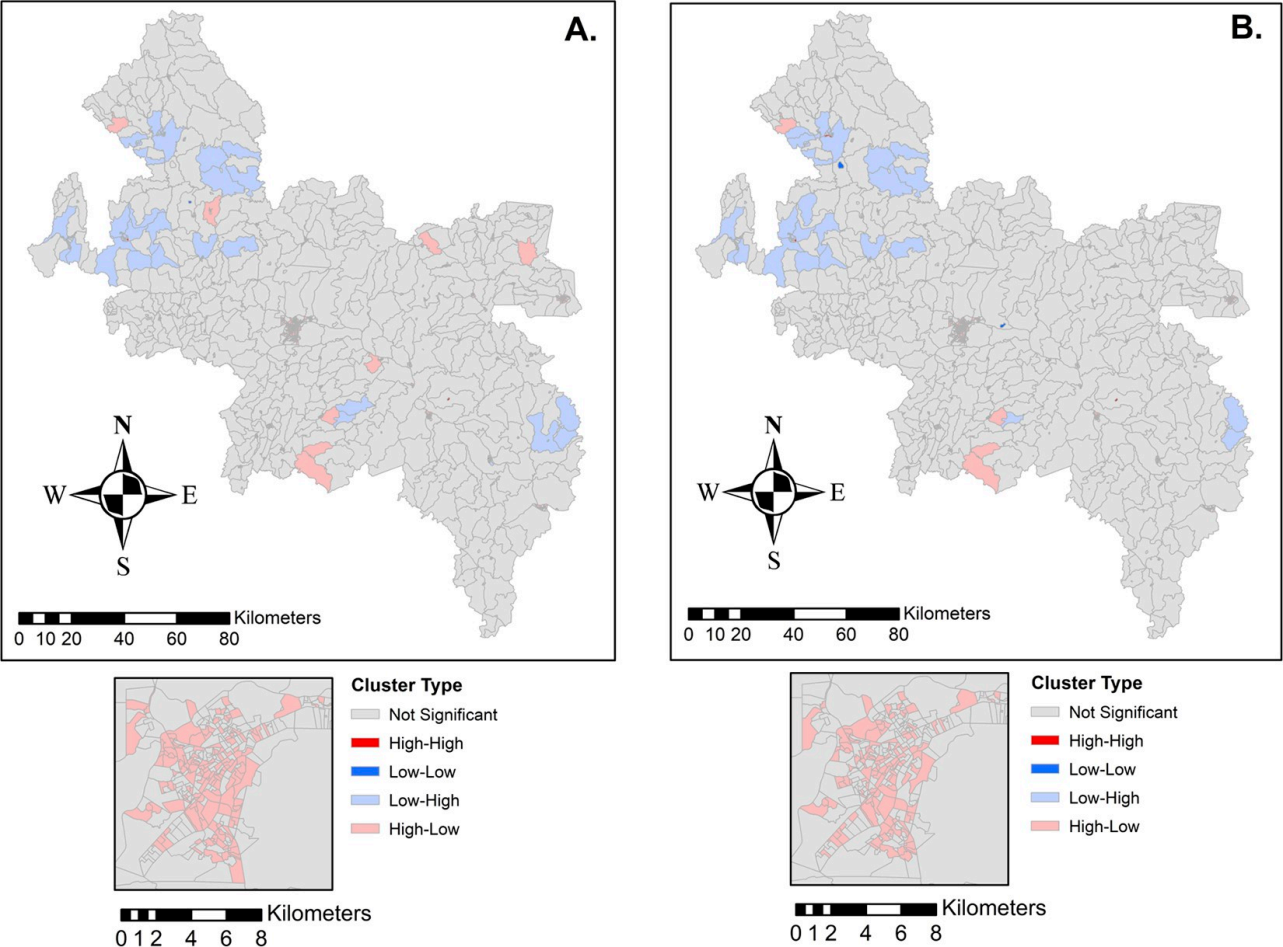
Bivariate Spatial Analysis: Bivariate LISA characterizing areas with a statistically significant (p<0.05) positive spatial association with the average annual incidence of HD and schistosomiasis (A) compared to MB disease and schistosomiasis (B). High-high clusters are small and difficult to visualize. Maps produced in ArcGIS 10.4 (ESRI, Redlands, CA, USA) using the spatial reference SIRGAS 2000 UTM Zone 24S30 (https://spatialreference.org/ref/epsg/31984/).

Overlap of schistosomiasis and HD occurred in 537 tracts, 28 of those tracts reporting MB disease, representing 2.9% and 2.2% of the total study area, respectively, but 10.4% of census tracts with HD present and 11.3% of MB present census tracts, respectively. Bivariate LISA detected significant associations (p<0.05) between the census tracts with high incidence rates of HD and schistosomiasis (high-high). For MB disease and schistosomiasis, eight areas of high-high clusters were identified. For all cases of HD with schistosomiasis, 13 high-high clusters were identified (**Fig 5**. and **Table 2**).

### Logistic regression census tract analysis

With the census tract as the unit of comparison, HD presence (yes or no) and MB presence (yes or no) were each modelled separately using multivariate logistic regression, with the main exposure of schistosomiasis (present in the census tract) and then controlled for household density (when significant or variable presence improved precision), population density, and average monthly census tract income. Controlling for all covariates, schistosomiasis presence was not significantly associated with total HD presence at the 5% significance level HD (aOR = 1.6, 95% CI = 1.0, 2.5) (**Table 4).** For HD, medium and high population density, as well as average and high average monthly income, were significantly associated (p<0.005, respectively). For the model looking only at MB Hansen’s disease, schistosomiasis presence was statistically significantly associated with MB disease with census tracts with incident schistosomiasis more likely to have incident MB (aOR = 1.7, 95% CI = 1.0, 2.7) (**Table 5).** Medium (aOR = 4.4, CI = 3.1, 6.2) and high (aOR = 5.2, CI = 3.6, 7.5) population density and average (aOR = 1.7, CI = 1.2, 2.1) and high (aOR = 2.8, CI = 1.7, 4.5) monthly income were also significantly associated with MB presence.

**Table 4:** Odds of Incident Hansen’s Disease Cases Per Census Tract With Schistosomiasis as Primary Exposure: Adjusted Odds Ratios and 95% confidence intervals presented.

Covariate	aOR (95% CI)
**Raw Data**	
**Schistosomiasis Presence**	
Yes	1.6 (1.0, 2.5)
**Population Density (people/km** ^**2**^ **)**	
<3000	REF
3000–6000	5.0 (3.5, 7.0)*
= >6000	7.1 (5.0, 10.1)*
**Average Monthly Income (Reais)**	
<600	REF
600–1200	1.6 (1.2, 2.2)*
= >1200	2.9 (1.8, 4.5)*

*p-value significant <0.05

**Table 5:** Odds of incident multibacillary Hansen’s disease cases per census tract with schistosomiasis as the primary exposure: adjusted odds Ratios and 95% confidence intervals, without spatial autocorrelation.

Covariate	aOR (95% CI)
**Schistosomiasis Presence**	
Yes	1.7 (1.0, 2.7)
No	REF
**Average Household Density, by census**	
<3	Ref
= >3	1.2 (0.8, 1.7)
**Population Density (people/km** ^**2**^ **)**	
<3000	REF
3000–6000	4.4 (3.1, 6.5)
= >6000	5.2 (3.6, 7.5)
**Average Monthly Income, by census**	
<600	REF
600–1200	1.7 (1.2, 2.4)
= >1200	2.8 (1.7, 4.5)

*p-value significant <0.05

### Census model global spatial autocorrelation

Incorporating spatial risk to the above model, positive spatial autocorrelation was significant for the final model for MB disease (Moran’s I = 0.14, p = 0.001). After comparing performance parameters, a spatial lag model was found to be the most improved upon model from the OLS model. Significant variables in the final MB disease model include schistosomiasis presence (aOR = 1.1, (95% CI 1.0, 1.2)), highest income (OR = 1.1, (95% CI 1.0, 1.3)), and medium (aOR = 1.2, (95% CI 1.1, 1.3)) and high (aOR = 1.2 (95% CI 1.1, 1.4)) population density. No spatial autocorrelation was detected in the final model for total HD cases (Moran’s I = 0.0003, p = 0.5).

## Discussion

Using both spatial and logistic regression modeling, we undertook the first of its kind geographic analysis of two co-endemic neglected tropical diseases of significant importance in Minas Gerais, Brazil. Given the utility of geographic information systems (GIS) in better understanding the distribution of neglected infections, our analyses present some interesting findings. Our data point to many areas of hyperendemic HD (40 cases / 100,000 annual case detection rate), with a high representation of MB disease, the more infectious form of HD (**Figs 2 and 3).** We also found clustering of leprosy and schistosomiasis individually as well as associations between the two infections (Tables 2 and 3). HD is often found in clusters, highlighting the importance of describing its epidemiology on fine resolution scales [20,22].

Clustering of HD was most notable in the census tracts of the major urban area of the study region, Governador Valadares, which may be explained by a higher population density in those areas. When investigating the most likely significant clusters (those that are most likely in Kulldorff’s spatial scan), there were two large clusters of both HD and MB (overlapping since MB is a subset of HD), with RR of HD or MB ranging 3–4, and highly statistically significant. This clustering suggests either ongoing person-to-person transmission in this area, shared risk factors for clinical disease, or common environmental reservoirs of infection. Another notable finding is that the average incidence is overall much higher in this region than the state overall (average of 8.71 per 100,000 in a similar time period), showing the high risk of Hansen’s disease in this region and the clusters revealed by the finer resolution at the census level [19.20].

Schistosomiasis also had some interesting findings by spatial scale. While HD was clustered in Governador Valadares, *S*. *mansoni* infections were clustered in areas outside of the city, with 43 high clusters and only 4 low clusters by LISA analysis. Since *S*. *mansoni* is transmitted from a snail intermediary by contact with fresh water, living in rural areas may therefore increase risk. And the clustering may suggest a shared source of infection or common risk factors. Two of the three most likely clusters (**Table 3**) of schistosomiasis showed a very high RR around 9, clearly showing the degree of spatial risk of this infection. The only published spatial analysis of schistosomiasis on a Minas Gerais state level was Fonseca et al., who estimated prevalence in eastern Minas Gerais using spatial regression models [4]. However, again, our resolution on the census tract again shows the finer details and heterogeneity within the endemic municipalities. This can give more useful data to public health authorities and control programs, who can target individual neighborhoods and nearby water sources.

When analyzing the two infections together, we did not see the extent of overlap that we saw in our small pilot study in Vespasiano, a single municipality where HD and schistosomiasis overlapped to a greater degree (RR 6.8, 95% CI 1.5–31.6) [15]. However, there were more high-high bivariate LISA clusters than low clusters of the combined infections (13 vs 3) which signals a potential interaction in the areas where the two diseases overlap. In addition, while the census tract odds of schistosomiasis were not statistically associated with total HD cases, there was a positive correlation with schistosomiasis and MB disease on the aspatial analysis with aOR = 1.7, 95% CI 1.0, 2.7. While incorporating spatial autocorrelation diminishes the association (aOR = 1.1, 95% CI 1.0, 1.2)) between MB and schistosomiasis, overall these results warrant further investigation, especially when taken with the results of the pilot study in Vespasiano [15]. The fact that MB distribution may be more related to schistosomiasis epidemiology than HD alone could suggest a mechanistic effect of co-infections–that in those with co-infection, schistosomiasis may predispose to the MB disease in those who develop disease. It has been proposed, with supporting evidence, that helminths, including schistosomiasis, can shift a host response, in the setting of co-infection away from a T-helper cell (Th1)-1 cell-mediated response due to the Th2 predominant response seen in helminths [7,37,38]. In HD, this could then result in either the development of clinical disease, the predisposition of multibacillary disease, or both [7,11,16]. Diniz et al. found an association with lepromatous HD and soil-transmitted helminths with a concomitant predominance of Th2 cytokines [7]. We have also shown an association with heminths (mostly schistosomiasis) and leprosy when cases were compared to household contacts [16]. In addition, Oktaria and all showed an association with MB disease and helminths [10] and many studies have demonstrated a Th2 immune shift in co-infections with helminths and *Mycobactrium tuberculosis* [39]. Some TB studies also support a risk of activation of latent TB in helminth infected individuals [39–41].

Therefore, given the biological plausibility and evidence to date, it is possible that in some of the overlapped areas, co-endemic schistosomiasis may be influencing the occurrence of multibacillary disease, the more infectious form of HD. While of course, direct co-infections cannot be inferred as the reason for these interactions in this ecological study, it does raise the possibility that co-infections may be at the root of this association. Alternatively, the presence of common risk factors for both MB Hansen’s disease and schistosomiasis may be a reason for this finding. As expected, we found that census tracts with higher population density were more likely to have HD and MB disease, although increased census tract income was unexpectedly associated with a higher risk of Hansen’s disease. However, overall, the mean income of 1200 reais per month was in fact, close to minimum wage, so none of the levels represented wealthy districts.

For a rare disease like HD, small variations in the number of cases can result in dramatic changes in disease rates, which could have impacted the interpretation of incidence. A spatially empirical Bayes (SEB) smoothed rate has been used in HD studies to smooth the random variations in small areas, such as census tracts [18,22,42]. Smoothing may enhance the visualization of spatial patterns as it did in our analyses where the distinction between regions with HD, MB disease, and schistosomiasis was more clearly defined. However, the smoothed data also had many more clusters. SEB can provide an estimation of suspected under-registration of a disease in a geographical area, addressing problems in underreporting of HD and schistosomiasis [34]. It also may help account for the lack of geocoded addresses in this study sample. However, caution of the interpretation of SEB is warranted as SEB does not take into account unrecognized differences between neighbors, such as geographic barriers or population fluctuations, and can inflate disease incidence towards local means where cases would not be seen [34].

There were several limitations to this analysis. Underreporting of both HD and *S*. *mansoni* infections could mask associations, especially in the case of schistosomiasis. Schistosomiasis can be asymptomatic and therefore, undiagnosed. It is also possible that some diagnosed cases go unreported since treatment is not dependent on reporting as it is in HD [43]. This underrepresentation could reduce the observed correlation with HD. Additionally, another limitation was that over 50% of the cases from SINAN, a passive surveillance system, did not report an address and could not be mapped to census tracts. Half of reported schistosomiasis cases and 30% of reported HD cases were missing and therefore, could not be spatially analyzed; however, the distribution of cases were not drastically different by municipality level between the full datasets and georeferenced subsets, possibly reducing the effect of this limitation (**S2 Fig**). Lastly, while HD reporting is compulsory, this is only done once cases are identified. One study in Bangladesh found that for every diagnosed case of HD there are 6 undiagnosed [44]. And a study in Brazil estimated that up to 10% of cases are not diagnosed or reported [21]. Therefore, it is possible that both the lack of addresses and underreporting could have either underestimate or overestimated the associations. Further work in spatial analyses of two or more infections is thus needed to better define these ecological associations and design more effective control strategies.

### Conclusions

We found clusters of HD and schistsosomiasis in eastern of Minas Gerais, Brazil, with some evidence of geographic overlap. Efforts should continue to map and spatially analyze cases using more accurate surveillance techniques, like active surveillance, to address limitations caused by passive surveillance. Environmental factors that could be associated with HD should also be incorporated into spatial studies to identify other risk factors for high endemicity. Finally, these results show an area of Brazil with ongoing transmission of leprosy and schistosomiasis, signaling a need for intensive control and management in order to achieve elimination.

## Supporting information

S1 Fig Data Flow Chart. (DOCX)

S2 Fig Distribution of Total HD Cases by Municipality, 2011–2015, Full (left) vs Geocoded (right) Datasets. Map produced in ArcGIS 10.4 (ESRI, Redlands, CA, USA) using the spatial reference SIRGAS 2000 UTM Zone 24S30" (https://spatialreference.org/ref/epsg/31984/)
(DOCX)

S3 Fig Distribution of Total Schistosomiasis Cases by Municipality, 2011–2015, Full (left) vs Geocoded (right. Map produced in ArcGIS 10.4 (ESRI, Redlands, CA, USA) using the spatial reference SIRGAS 2000 UTM Zone 24S30" Datasets (https://spatialreference.org/ref/epsg/31984/)
(DOCX)

S4 Fig Bivariate K-function (MB disease vs PB disease). (DOCX)
