# Supplementary figures and images for: Spatial associations of Hansen’s disease and schistosomiasis in endemic regions of Minas Gerais, Brazil
Source: PLoS Negl Trop Dis. 2024 Dec 26;18(12):e0012682. doi: 10.1371/journal.pntd.0012682 (PMC11753700; doi:10.1371/journal.pntd.0012682)

# **S1 Fig**. Data Flow Chart


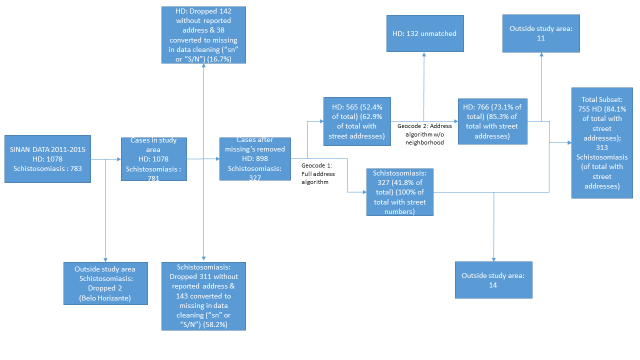

Supplement: S1 Fig — (DOCX) [file pntd.0012682.s001.docx]

## **S2 Fig.** Distribution of HD Cases by Municipality, 2011-2015, Full vs Geocoded Data


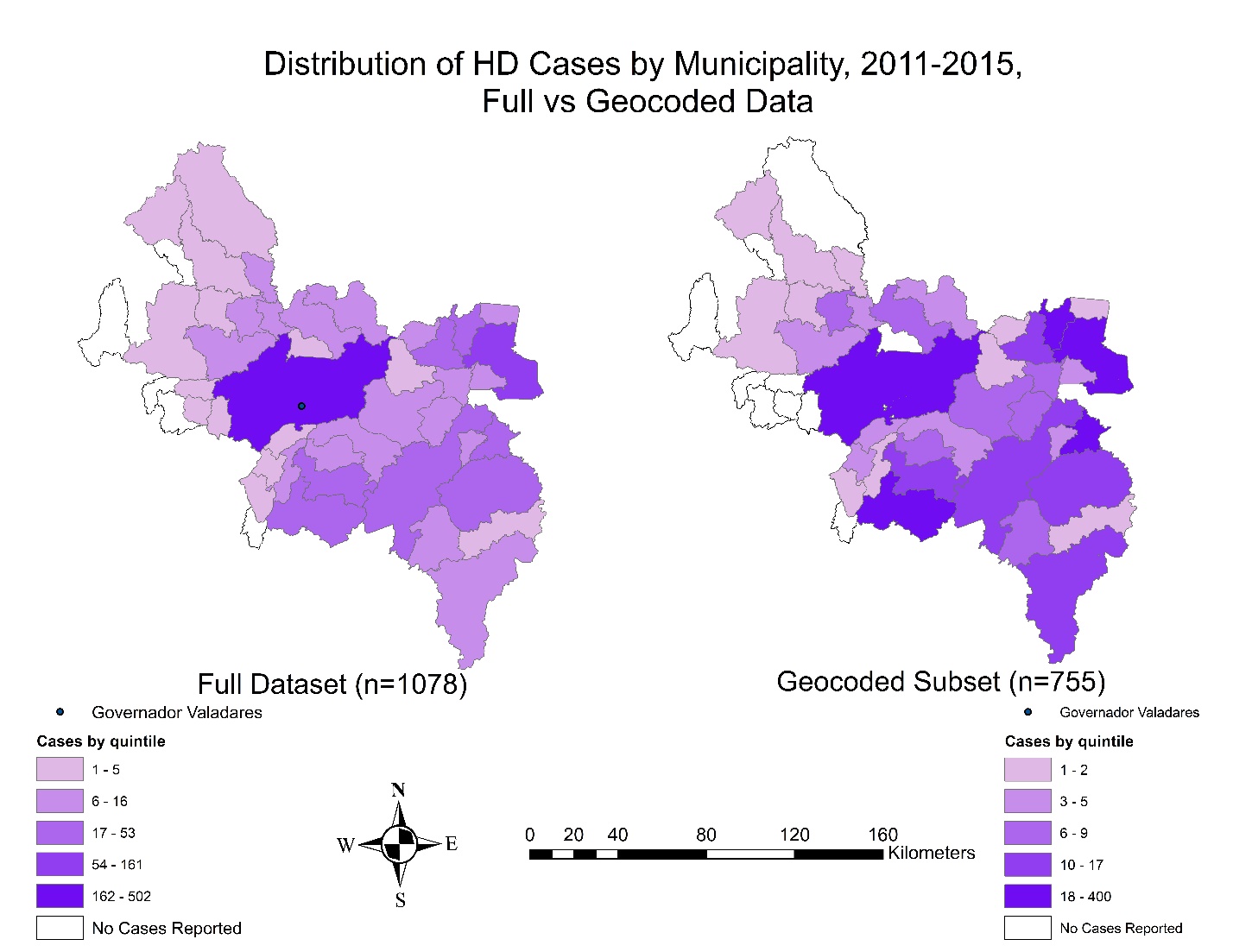

Supplement: S2 Fig — Distribution of Total HD Cases by Municipality, 2011–2015, Full (left) vs Geocoded (right) Datasets. Map produced in ArcGIS 10.4 (ESRI, Redlands, CA, USA) using the spatial reference SIRGAS 2000 UTM Zone 24S30" (https://spatialreference.org/ref/epsg/31984/) (DOCX) [file pntd.0012682.s002.docx]

**S3 Fig.** Distribution of Schistosomiasis Cases by Municipality, 2011-2015, Full vs Geocoded Data


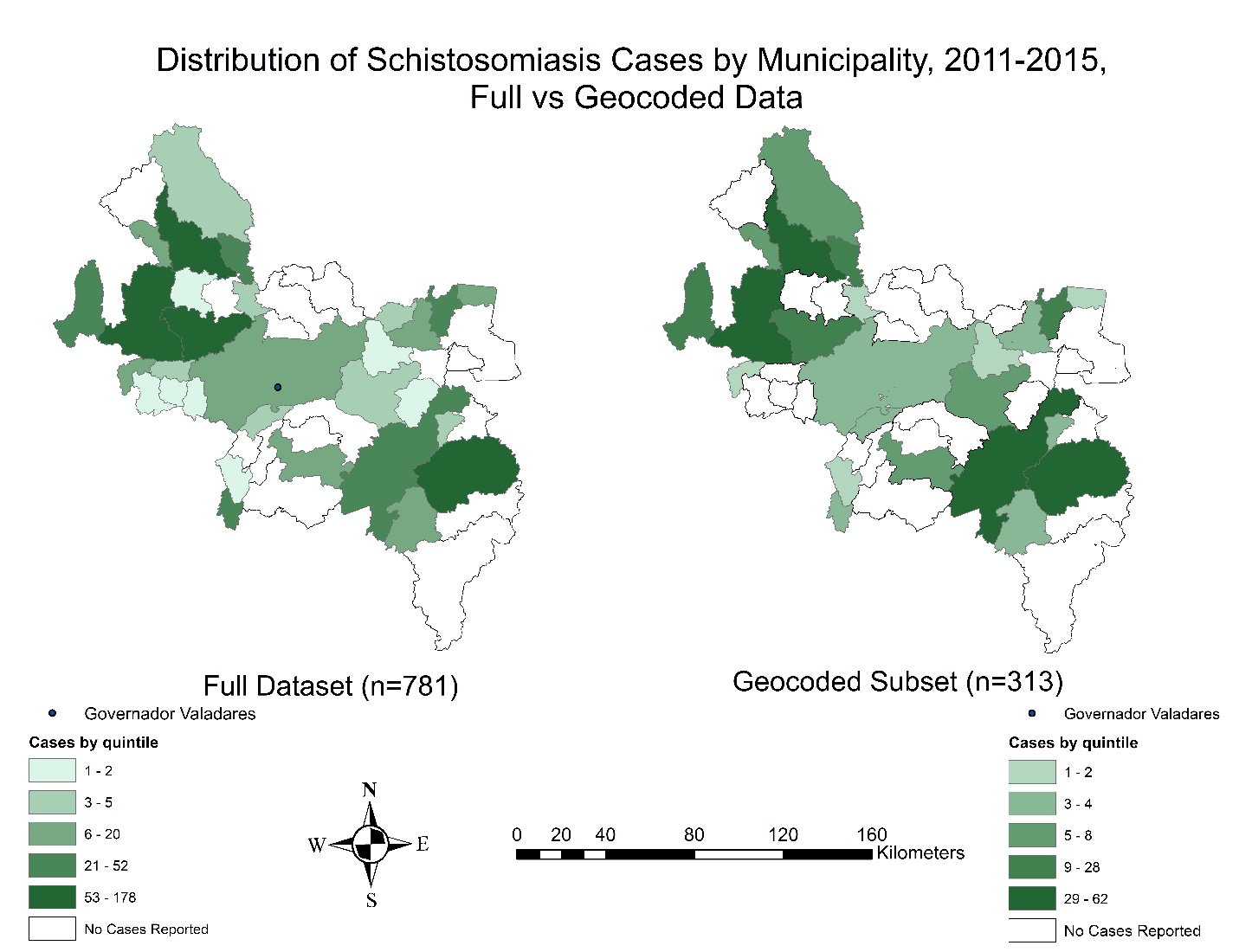

Supplement: S3 Fig — Distribution of Total Schistosomiasis Cases by Municipality, 2011–2015, Full (left) vs Geocoded (right. Map produced in ArcGIS 10.4 (ESRI, Redlands, CA, USA) using the spatial reference SIRGAS 2000 UTM Zone 24S30" Datasets (https://spatialreference.org/ref/epsg/31984/) (DOCX) [file pntd.0012682.s003.docx]
