# Supplementary material for: Spatial associations of Hansen’s disease and schistosomiasis in endemic regions of Minas Gerais, Brazil
Source: PLoS Negl Trop Dis. 2024 Dec 26;18(12):e0012682. doi: 10.1371/journal.pntd.0012682 (PMC11753700; doi:10.1371/journal.pntd.0012682)
Supplement: S4 Fig — (DOCX) [file pntd.0012682.s004.docx]

## **S4 Fig.** Bivariate K-function (MB disease vs PB disease)
